# Conventional Housing Conditions Attenuate the Development of Experimental Autoimmune Encephalomyelitis

**DOI:** 10.1371/journal.pone.0099794

**Published:** 2014-06-11

**Authors:** Andreas Arndt, Peter Hoffacker, Konstantin Zellmer, Oktay Goecer, Mascha S. Recks, Stefanie Kuerten

**Affiliations:** 1 Department of Anatomy I, University of Cologne, Cologne, Germany; 2 Department of Anatomy and Cell Biology, University of Wuerzburg, Wuerzburg, Germany; Julius-Maximilians-Universität Würzburg, Germany

## Abstract

**Background:**

The etiology of multiple sclerosis (MS) has remained unclear, but a causative contribution of factors outside the central nervous system (CNS) is conceivable. It was recently suggested that gut bacteria trigger the activation of CNS-reactive T cells and the development of demyelinative disease.

**Methods:**

C57BL/6 (B6) mice were kept either under specific pathogen free or conventional housing conditions, immunized with the myelin basic protein (MBP)–proteolipid protein (PLP) fusion protein MP4 and the development of EAE was clinically monitored. The germinal center size of the Peyer’s patches was determined by immunohistochemistry in addition to the level of total IgG secretion which was assessed by ELISPOT. ELISPOT assays were also used to measure MP4-specific T cell and B cell responses in the Peyer’s patches and the spleen. Ear swelling assays were performed to determine the extent of delayed-type hypersensitivity reactions in specific pathogen free and conventionally housed mice.

**Results:**

In B6 mice that were actively immunized with MP4 and kept under conventional housing conditions clinical disease was significantly attenuated compared to specific pathogen free mice. Conventionally housed mice displayed increased levels of IgG secretion in the Peyer’s patches, while the germinal center formation in the gut and the MP4-specific T_H_17 response in the spleen were diminished after immunization. Accordingly, these mice displayed an attenuated delayed type hypersensitivity (DTH) reaction in ear swelling assays.

**Conclusions:**

The data corroborate the notion that housing conditions play a substantial role in the induction of murine EAE and suggest that the presence of gut bacteria might be associated with a decreased immune response to antigens of lower affinity. This concept could be of importance for MS and calls for caution when considering the therapeutic approach to treat patients with antibiotics.

## Introduction

Multiple sclerosis (MS) is considered to be an autoimmune disorder of the central nervous system (CNS) and the most common cause of irreversible disability in young adults [1]. The etiology of MS has remained unclear, but one of the currently discussed hypotheses emphasizes the potential role of commensal gut bacteria [2]–[4]. It has been shown that molecular mimicry between known autoimmune immunogens such as myelin basic protein (MBP) or oligodendrocyte glycoprotein (MOG) and nonpathogenic gut bacteria frequently occurs [5] and could explain the autoimmune activity in MS patients. Among these potential bacteria resembling CNS antigens are *E. coli*, *Lactobacillus spp.*, *Enterobacter* and *Streptococcus spp*. [5]. Experimental autoimmune encephalomyelitis (EAE) is a common animal model of MS. EAE is induced by active immunization with CNS antigens [6] that are typically derived from the myelin sheath, or by the passive transfer of autoreactive T cells [7]. In addition, spontaneous models exist that make use of genetically-modified mice, which harbor T cell receptor (TCR) transgenic T cells [8]–[13]. Studying a spontaneous model of EAE that involves MOG: 92–100 transgenic T cells, it has been shown that germ-free mice did not develop EAE compared to mice that were housed in a specific pathogen free (SPF) environment [2]. The authors suggested that autoreactive T cells were activated by commensal microbiota in the gut, where the cells expanded and subsequently triggered autoantibody production by B cells [2]. This concept is clinically highly relevant since non-invasive therapeutic strategies such as the administration of antibiotics to MS patients represent a reasonable option with low risks for adverse side effects. Indeed, reports exist that explored the impact of antibiotic treatment in EAE. The oral treatment of SJL and C57BL/6 (B6) mice with broad spectrum antibiotics resulted in the attenuation of the disease, which was associated with decreased levels of proinflammatory cytokines and an increase in IL-10 and IL-13 production [14].

In the present study we demonstrate that EAE was significantly attenuated in mice when kept under conventional housing conditions compared to specific pathogen free mice. While SPF mice developed severe EAE, conventionally kept mice frequently showed no disease or a mild/transient disease course. We employed a mouse model of EAE, in which disease was actively induced by immunization of B6 mice with the MBP/proteolipid protein (PLP) fusion protein MP4 and that was recently introduced by our group [15]. We chose this model because of its dependence on both B cells/antibodies and T cells [15], [16], thus covering the immunology of MS more closely than the traditional EAE models that are focused on peptide-specific T cell responses.

Next to a potential cross-reactivity between gut microbiota and T cells and the induction of a pro-inflammatory microenvironment, we propose antigenic competition as an additional mechanism that could be involved in the disease. Accordingly, the high-affinity immune response triggered by gut bacteria might compete with the low-affinity self-antigen response against CNS antigens, which is subsequently diminished. The data presented in the following evolve around this hypothesis and imply that gut bacteria could play both a protective and harmful role in the pathogenesis of MS.

## Materials and Methods

### Mice

B6 mice were purchased from Harlan Laboratories (Rossdorf, Germany) and bred under SPF or conventional conditions under a 12 h light/dark cycle at the animal facilities of the Department of Anatomy, University of Cologne. Mice were screened according to the FELASA health monitoring recommendations. SPF mice displayed infection with *Streptococci* in the gastrointestinal tract, while conventionally housed mice showed the presence of *E. coli*, *Enterococcus spp.*, *Staphylococcus spp., Lactobacillus spp.* and *Streptococci*. All mice were 6–8 weeks old at the time of immunization. All animal experiments complied with the German Law on the Protection of Animals and the “Principles of laboratory animal care” (NIH publication No. 86–23, revised 1985). All treatments were performed according to a protocol that was approved by the LANUV, Germany (approval number 2011.A276). This protocol included that all animals were monitored daily for clinical signs of EAE and in the case of EAE received easier access to food and water. Animals displaying an EAE score greater than 3 for more than three consecutive days and/or weight loss that was greater than 15% within a few days or gradually greater than 20% were sacrificed. All mice were sacrificed using CO_2_. Overall, n = 21 SPF MP4-immununized, n = 15 SPF age-matched controls, n = 25 conventionally kept MP4-immunized and n = 17 age-matched conventionally kept control mice were included in the analyses. Control mice were not immunized. Both male and female mice were studied.

### EAE Induction

The MBP-PLP fusion protein MP4/Apogen (containing the 21.5 kD isoform of human MBP and the three hydrophilic domains of PLP) was obtained from Alexion Pharmaceuticals (Cheshire, CT). Incomplete Freund’s adjuvant (IFA) was prepared as a mixture of mannide monooleate (Sigma-Aldrich, St. Louis, MO) and paraffin oil (EMScience, Gibbstown, NJ). CFA was obtained by mixing *Mycobacterium tuberculosis* H37 Ra (Difco Laboratories, Franklin Lakes, NJ) at 5 mg/ml into IFA. For active immunization, B6 mice were immunized subcutaneously in both sides of the flank with a total dose of 200 µg MP4 in CFA. Pertussis toxin (List Biological Laboratories, Hornby, ONT, Canada) was given at 200 ng per mouse on the day of immunization and 48 h later. Clinical assessment of EAE was performed daily according to the following criteria: (0), no disease; (1), floppy tail; (2), hind limb weakness; (3), full hind limb paralysis; (4), quadriplegia; (5), death. Mice that were in between the clear-cut gradations of clinical signs were scored intermediate in increments of 0.5.

### Histology and Immunohistochemistry (IHC)

Mice were sacrificed 23–30 days after immunization with MP4 using CO_2_. The Peyer’s patches were carefully dissected and snap-frozen in liquid nitrogen. The tissue was stored at −80°C until analysis. Seven µm thick sections were cut on a cryostat. The frozen sections were air-dried and post-fixed in paraformaldehyde (PFA) (Serva Electrophoresis GmbH, Heidelberg, Germany). Sections were washed with PBS +0.05% TWEEN 20 and blocked with 3% bovine serum albumin (BSA) (PAA, Pasching, Austria) and 5% normal mouse serum (Vector Laboratories, Burlingame, CA, USA) in PBS for 1 h. Sections were then incubated with the primary antibodies directed against Ki67 (abcam, Cambridge, UK; diluted 1∶1000), MAdCAM-1 (BD Biosciences, Heidelberg, Germany; diluted 1∶200), B220 (eBioscience, Frankfurt, Germany; diluted 1∶500), TCR (BD Biosciences; diluted 1∶200), CD35 (BD Biosciences; diluted 1∶200) or Bcl-6 (Santa Cruz Biotech, Dallas, TX; diluted 1∶100) in blocking solution at 4°C overnight. The anti-TCR antibody was biotinylated. Endogenous peroxidase activity was blocked with 50% methanol und 1.66% H_2_O_2_. Sections were incubated with secondary biotin-conjugated rabbit anti-rat (1∶250), rabbit anti-goat (1∶250) or goat anti-rabbit antibodies (1∶400) (Dako, Hamburg, Germany) in blocking solution for 2 h at RT. After renewed washing, extravidine-peroxidase (Dako) (1∶100 in PBS +0.05% TWEEN 20) was added for 1 h. Sections were washed again and developed with filtered DAB solution (containing 7.5 mg DAB, 6 mg NH_4_Cl, 0.05 M NiSO_4_, 0.2% glucose, 0.004 g glucose oxidase in PB pH = 7.4) for 2–10 min. Sections were rinsed with distilled water, and incubated with methyl green (Vector Laboratories; diluted 1∶3 in distilled water) to counterstain cellular nuclei. Sections were again washed with distilled water and overlaid with isopropanol in order to dry them. Sections were rinsed in xylene and coverslipped with Entellan. Sections were observed with a Leica DM LB2 microscope and digital images were acquired with an AxioCam (Zeiss, Oberkochen, Germany) and Zeiss software (AxioVision 40 4.7). For the B220/TCR IHC sections were incubated with Neutravidin Dylight 488 and 550 (Thermo Scientific, Bonn, Germany). Counterstaining of cellular nuclei in fluorescence stainings was done with Hoechst 33342 (Thermo Scientific) (1∶1000 in washing solution). Fluorescent sections were analyzed on a Zeiss Axioskop 50 epifluorescence microscope using Carl Zeiss Plan-NEOFLUAR 109/0.30 and 409/1.30 objectives. For fluorescence analysis Carl Zeiss filter sets No. 1 (excitation BP 365/12, emission LP 397) and No. 15 (excitation BP 546/12, emission 590 nm) were used, respectively. Digital images were acquired using a Leica DFC350FX camera and software.

### Evaluation of Germinal Centers

Germinal center formation was measured in two sections of each Peyer’s patch per mouse. One image was acquired per Peyer’s patch at 4x to measure the size of the Peyer’s patch in addition to the size of the germinal center. All sections were observed with a Leica DM LB2 microscope. Digital images were acquired with an AxioCam (Zeiss) and Zeiss software (AxioVision 40 4.7), which was also used for all measurements.

### Enzyme-Linked Immunospot Assays (ELISPOT)

T cell ELISPOT assays were performed on days 9–15 and B cell ELISPOT assays on days 23–30 after immunization. The spleen or inguinal and mesenteric lymph nodes as well as the Peyer’s patches were removed, disintegrated mechanically and filtered through a 70 µm nylon cell strainer (BD Falcon, Heidelberg, Germany). After washing the cells with RPMI-1640 (Biochrom AG, Berlin, Germany) and counting them with acridine orange (0.1%, Sigma)/ethidium bromide (0.1%, Serva Electrophoresis), cells were resuspended in HL-1 (Lonza, Cologne, Germany) supplemented with 1% L-glutamine (Sigma) and 1% penicillin/streptomycin (Sigma). ELISPOT plates (Merck KGaA, Darmstadt, Germany) were coated overnight with the capture antibodies rat anti-mouse interferon (IFN)-γ (final concentration 3 µg/ml, clone AN-18; eBioscience, Frankfurt, Germany) and rat anti-mouse interleukin (IL)-17 (final concentration 2 µg/ml, clone eBio17CK15A5; eBioscience) in PBS. For B cell ELISPOT assays plates were coated with PBS (negative control), MP4 (at 10 µg/ml) or anti-IgG (positive control) (at 15 µg/ml; Mabtech, Nacka Strand, Sweden). Plates were washed with PBS, and blocked with 1% BSA (PAA) in PBS for T cell assays or 10% FBS (Gibco, Darmstadt, Germany) in PBS for B cell assays for 2 h at room temperature. Cells were plated at 1–2×10^6^ cells/well. For T cell assays cells were incubated either with medium, MP4 (final concentration: 30 µg/ml) or anti-CD3 (final concentration: 3 µg/ml; clone 2C11, kindly provided by M. Tary-Lehmann) at 7% CO_2_ and 37°C for 24 h. Plates were washed and incubated with biotin-conjugated anti-IFN-γ (1 µg/ml; eBioscience, clone R4-6A2) or anti-IL-17 (1 µg/ml; eBioscience, clone eBio17B7) overnight at 4°C. B cell ELISPOT plates were incubated with biotin-conjugated IgG (Dako, diluted 1∶2000 in 0.5% FBS/PBS) overnight at 4°C. The next day, plates were washed and incubated with streptavidin-conjugated alkaline phosphatase (Dako; diluted 1∶1000). After renewed washing, plates were developed with Vector Blue (Vector Laboratories) according to the manufacturer’s instructions. Plates were washed with distilled water and finally dried overnight. Spots were counted with an ImmunoSpot Series 6 UV Analyzer (Cellular Technology Limited, Shaker Heights, OH). All results were medium-subtracted and normalized to 10^6^ cells per well.

### DTH Assay

On days 22–25 after immunization, mice were challenged with 10 µg of soluble antigen injected intradermally into the ear. One ear was injected with MP4 while the other was injected with ovalbumin (OVA) as irrelevant antigen. Alternatively, mice were either challenged with MP4 or OVA injecting both ears with the same antigen. Twenty-four hours postchallenge, ear biopsies were taken, fixed in 4% PFA and paraffin embedded. Sections were stained with haematoxylin and eosin and observed with a Leica DM LB2 microscope. Digital images were acquired with an AxioCam (Zeiss) and Zeiss software (AxioVision 40 4.7). One image was acquired per ear at 10x for measuring the extent of ear swelling. Three images were acquired per ear at 40x for the evaluation of the amount of infiltrating leukocytes per mm^2^.

### Statistical Analysis

Differences between groups were assessed by Student’s *t*-test. In case the Normality Test or the Equal Variance Test failed, the Mann-Whitney U rank-sum test was used (calculated by SigmaPlot, version 12.0, SPSS Inc., Chicago, IL). Statistical significance was set at *p*≤0.05.

## Results

### The Development of EAE is Significantly Attenuated in Mice Kept under Conventional Conditions

In order to investigate the contribution of commensal gut bacteria to the development of EAE, we induced active EAE in two groups of B6 mice kept under different housing conditions. While one group (n = 8) was kept under SPF conditions in isolated-ventilated cages and received autoclaved water and feed, the other group (n = 10) was maintained in conventional open cages with non-autoclaved feed and tap water. Mice from both groups were immunized with 200 µg MP4 in complete Freund’s adjuvant (CFA) and received injections of pertussis toxin on days 0 and 2. The clinical course of the disease in both groups was assessed over a period of 80 days. Mice kept under SPF conditions developed clinical EAE as early as day 10 (mean 12.1±3.1) after immunization, while mice kept under conventional conditions showed first paralytic signs on day 13 (mean 18.4±4.6) after immunization (*p* = 0.009, rank-sum test). In the SPF group all mice developed EAE, compared to 7 of 10 conventionally housed mice. In addition, disease severity was considerably reduced in mice kept under conventional conditions with a mean EAE score of 0.50±0.27 compared to 1.99±0.27 in mice kept under SPF conditions (*p*<0.001, rank-sum test).

### Histological Characterization of the Peyer’s Patches in the Gut

**Figure 1:**
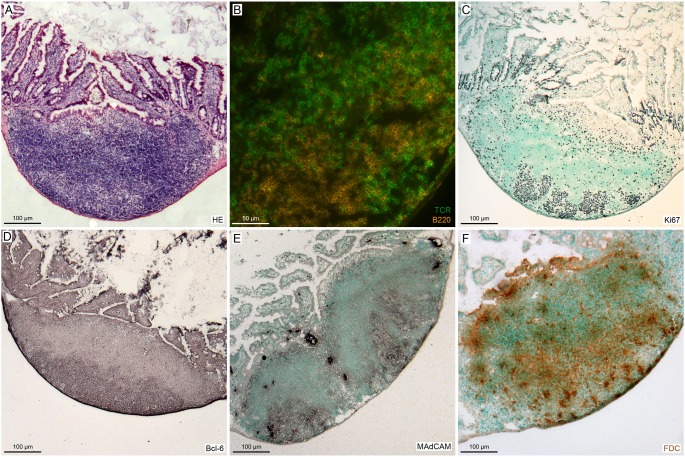
Histological characterization of the Peyer’s patches. Mice were housed either under conventional or SPF conditions and actively immunized with 200 µg MP4. On days 23–30 after immunization the Peyer’s patches were studied histologically. A, Haematoxylin/eosin staining. B, IHC staining for B cells (B220^+^) and T cells (TCR^+^). C, Ki67 staining for proliferation. D, Staining of the germinal center marker Bcl-6. E, Presence of high endothelial venules expressing MAdCAM-1 within the Peyer’s patches. F, Presence of follicular dendritic cells. The images are representative for a total of n = 6 SPF and n = 6 conventionally housed MP4-immunized mice.

To assess the immunological response in the gut induced by immunization with MP4, histological sections were obtained from all Peyer’s patches of n = 6 conventionally and n = 6 SPF housed mice. The gut was removed after mice had been sacrificed and the Peyer’s patches were localized macroscopically. Each Peyer’s patch was carefully excised, placed in freezing medium and snap-frozen in liquid nitrogen. To ensure that the excised tissue specimen indeed contained Peyer’s patches we stained representative sections for features that are characteristic of Peyer’s patches. Results are shown in Figure 1. The Peyer’s patches were characterized by the presence of T and B cell zones (Figure 1B), germinal center markers such as Ki67 indicating proliferation (Figure 1C) and Bcl-6 (Figure 1D), high endothelial venules expressing MAdCAM-1 (Figure 1E) as well as follicular dendritic cells (FDC) (Figure 1F).

### Mice Kept under SPF Conditions Show an Increased Germinal Center Reaction in Peyer’s Patches after Immunization

**Figure 2:**
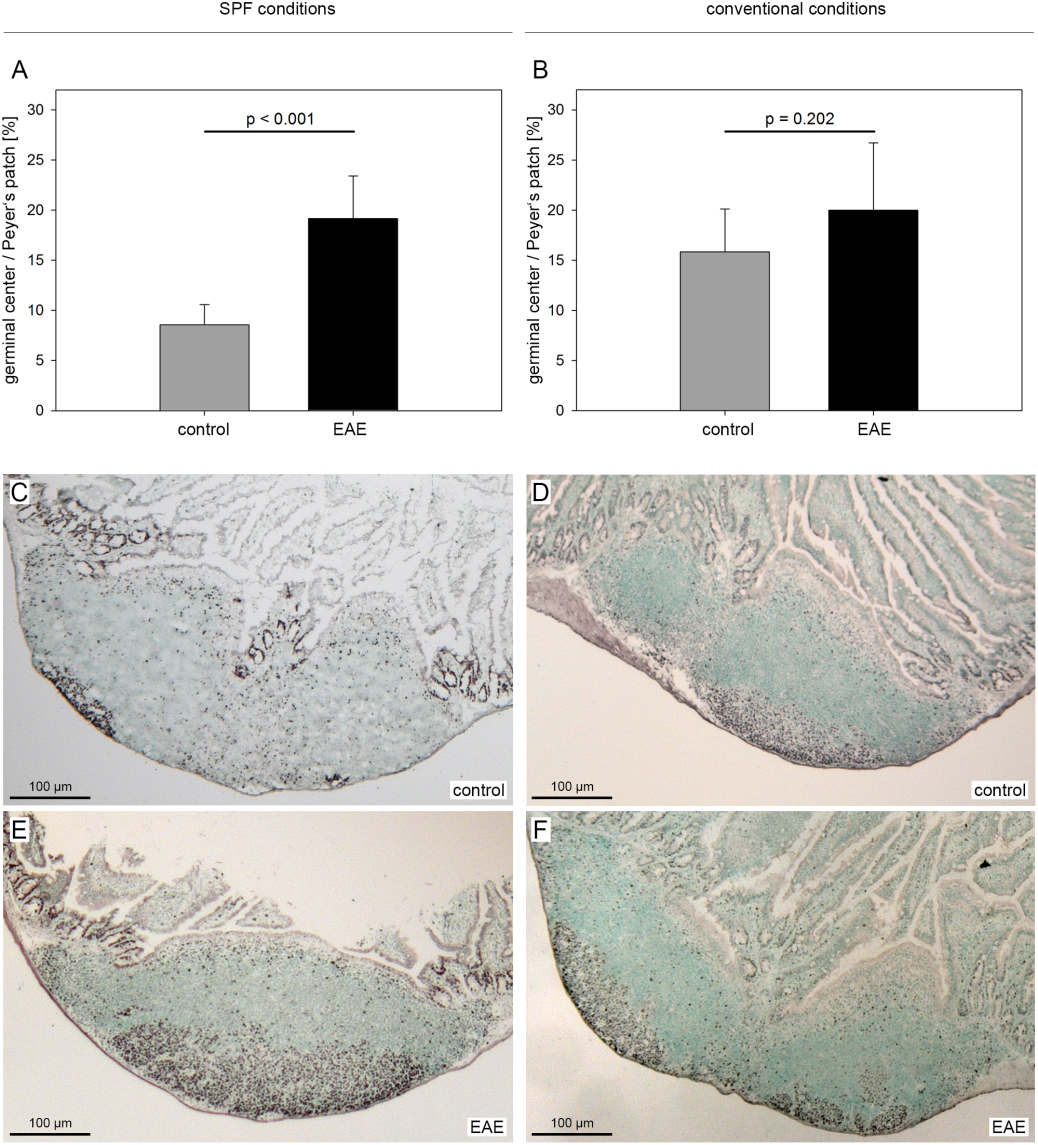
Increased germinal center reactions in Peyer’s patches after immunization in SPF housed mice. Mice were housed either under conventional or SPF conditions and actively immunized with 200 µg MP4. On days 23–30 after immunization the germinal center size was studied histologically on Ki67-stained sections taken from the Peyer’s patches of n = 6 conventionally and n = 6 SPF housed mice. In addition n = 7 conventionally and n = 9 SPF housed unimmunized control mice were studied. A, Germinal center size per Peyer’s patch area shown as mean + SD in SPF mice. B, Germinal center size per Peyer’s patch area shown as mean + SD in conventionally housed mice. C–F, Representative images of Ki67 IHC in the different groups.

B6 mice were kept either under conventional or SPF conditions and immunized with MP4 as described in *Materials and Methods*. On day 23–30 after immunization when the MP4-induced B cell response had reached its maximum [17] the gut was removed and sections were obtained from the Peyer’s patches of n = 6 conventionally housed and n = 6 SPF mice. In addition, sections were obtained from n = 7 conventionally housed and n = 9 SPF age- and gender-matched control mice. Sections were stained with the proliferation marker Ki67 reflecting the germinal center size. Alternative staining was done using the germinal center marker Bcl-6 with identical results. Results are shown in Figure 2. SPF control mice displayed a mean germinal center size of 8.6±2.0% in relation to the total Peyer’s patch area. After immunization the size was significantly increased reaching a value of 19.2±4.2% (*p*<0.001, Student’s *t*-test). In conventionally housed mice the germinal center size at baseline was elevated to the level of the immunized SPF mice (15.8±4.3%). The size did not show any further significant increase after immunization (20.0±6.7%, *p* = 0.202; Student’s *t*-test).

### Increased IgG Secretion in Peyer’s Patches of Mice Kept under Conventional Conditions

**Figure 3:**
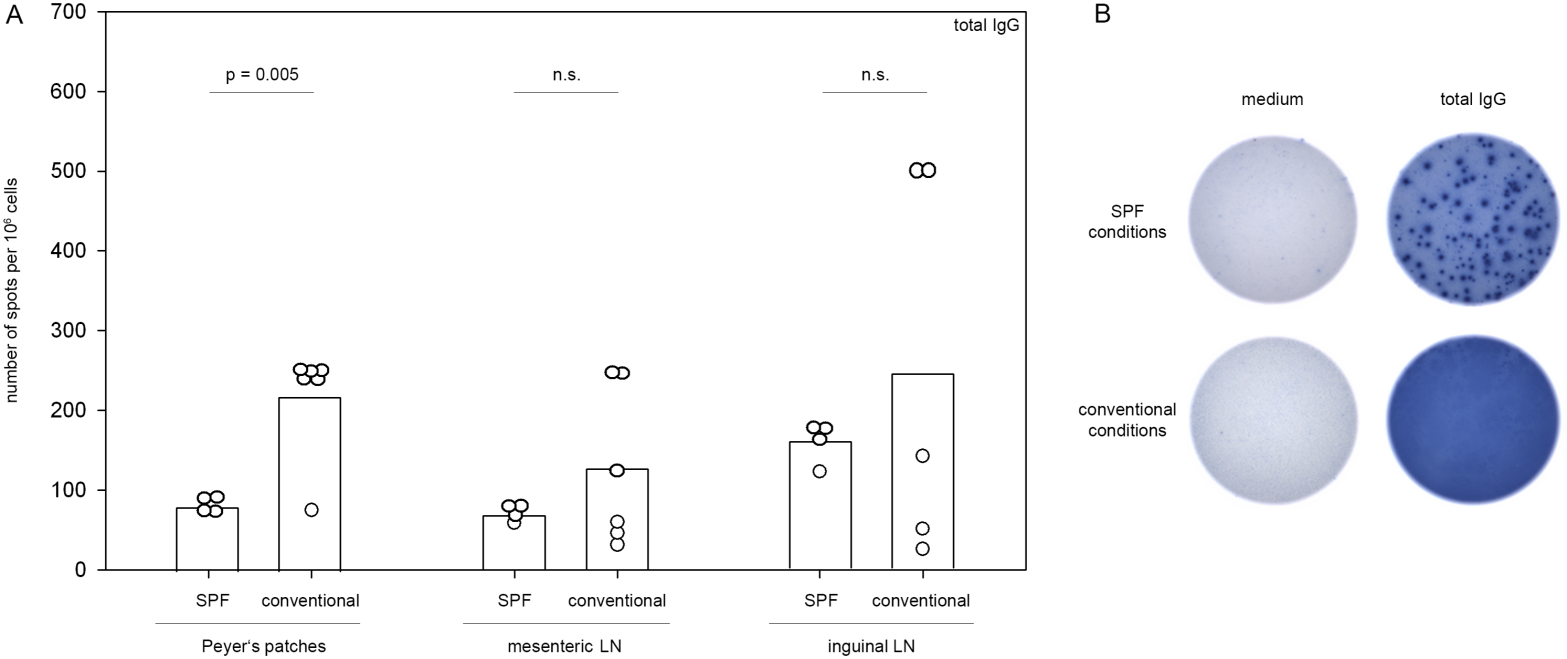
Increased IgG secretion in Peyer’s patches of mice kept under conventional conditions. Mice were housed either under conventional (n = 6) or SPF (n = 4) conditions and actively immunized with 200 µg MP4. On days 23–30 after immunization ELISPOT assays were performed to measure the extent of total IgG secretion in the Peyer’s patches. A, Number of spots per 10^6^ plated cells derived from the Peyer’s patches, the mesenteric or inguinal lymph nodes (LN). B, Representative ELISPOT well images showing medium control and total IgG responses in SPF and conventionally housed mice.

**Figure 4:**
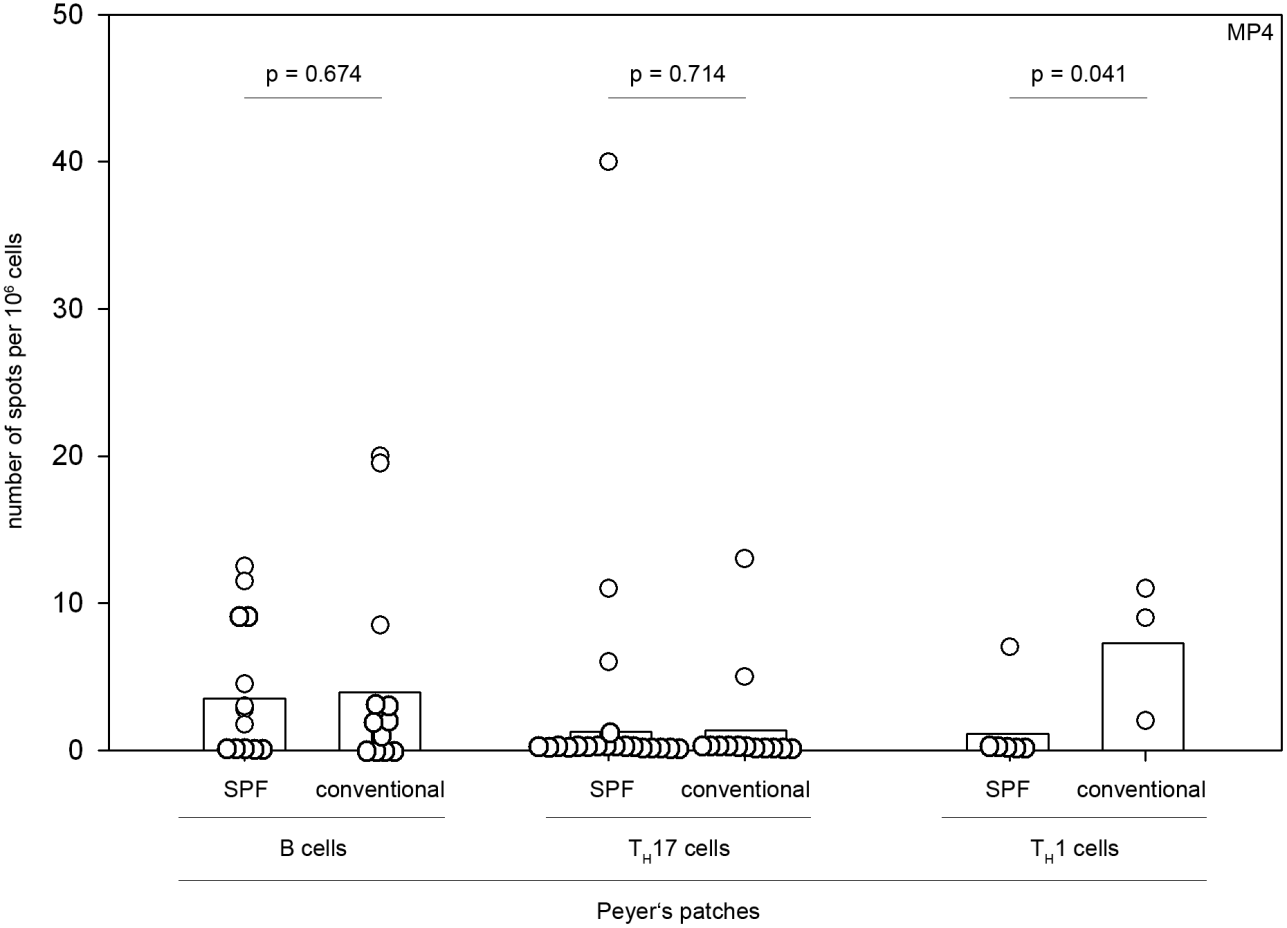
MP4-specific production of IgG, IL-17 and IFN-γ in the gut. Mice were housed either under conventional or SPF conditions and actively immunized with 200 µg MP4. On days 23–30 after immunization ELISPOT assays were performed to measure the MP4-specific B cell response in the Peyer’s patches (n = 14 mice in each group). On days 9–15 after immunization ELISPOT assays were performed to measure the MP4-specific secretion of IL-17 (n = 19 SPF and n = 13 conventionally housed mice) and IFN-γ (n = 6 SPF versus n = 3 conventionally housed mice) in the Peyer’s patches. Data are shown for individual mice represented by circles. The mean values are shown by the bars.

To test the hypothesis that an increased baseline germinal center size in conventionally housed mice was reflective of a generally elevated immune response in the Peyer’s patches we additionally performed ELISPOT analysis testing for total IgG secretion after immunization. Results were compared to measurements of the total IgG secretion in the draining mesenteric and inguinal lymph nodes. As shown in Figure 3 conventionally housed mice (n = 6) displayed significantly elevated levels of total IgG secretion in the Peyer’s patches compared to SPF mice (n = 4) (218.33±70.39 spots per 10^6^ cells versus 80.13±7.48 spots/10^6^ cells, *p* = 0.005; Student’s *t*-test) while results for the mesenteric and inguinal lymph nodes were comparable. Notably, there was no difference in the immune response in the Peyer’s patches directed against MP4. In both SPF and conventionally housed mice a MP4-specific B cell and/or T cell response was only detectable in the minority of mice by ELISPOT assays measuring MP4-specific IgG, IL-17 or IFN-γ secretion (Figure 4). The magnitude of the MP4-specific IgG, IL-17 and IFN-γ secretion was low and comparable between the two groups (Figure 4).

### The T_H_17-mediated Delayed Type Hypersensitivity (DTH) Reaction Was Significantly Attenuated in Mice Kept under Conventional Conditions

**Figure 5:**
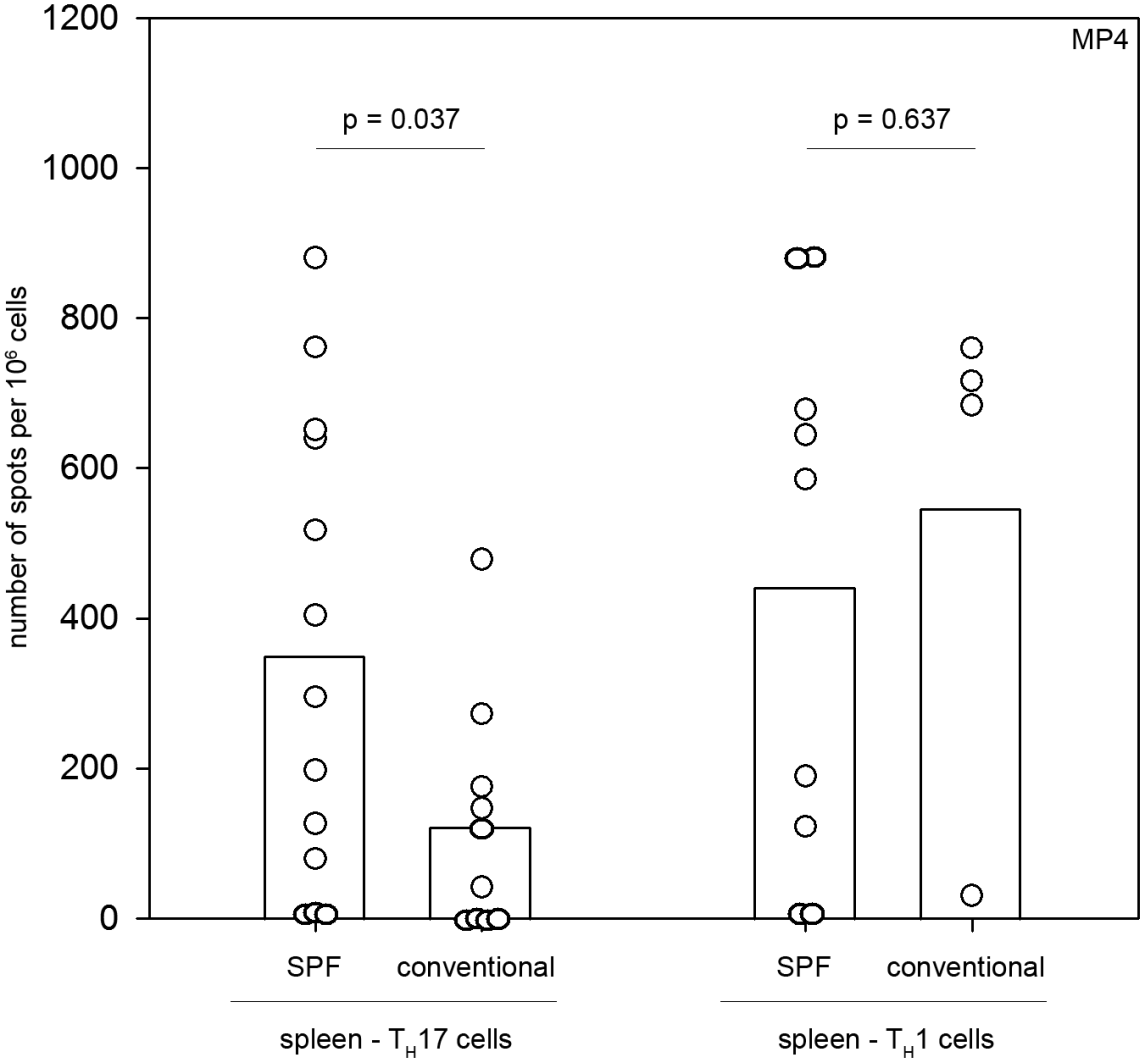
Attenuated MP4-specific IL-17 production in conventionally housed mice. Mice were housed either under conventional (n = 10) or SPF (n = 13) conditions and actively immunized with 200 µg MP4. On days 9–15 after immunization ELISPOT assays were performed to measure the MP4-specific secretion of IFN-γ and IL-17 in the spleen. Data are shown for individual mice that are represented by circles. The mean values are shown by the bars.

An elevated immune response triggered by gut bacteria in conventionally housed mice could have a major impact on the MP4-specific response and explain the attenuated disease development. We measured the magnitude of the MP4-specific IL-17 response in the spleens of MP4-immunized mice between days 9–15 by ELISPOT. We focused on measurements of IL-17 because the encephalitogenic capacity of CFA to induce disease has been shown to be linked to T_H_17 cells [18] and this cell type has been implicated both in mediating autoimmune pathology and eliciting DTH reactions [19]–[21]. As demonstrated by Figure 5 the MP4-induced IL-17 response was significantly diminished in conventionally housed mice after MP4 immunization (123.4±155.9 spots/10^6^ cells in conventionally housed mice (n = 10) compared to 350.6±311.72 spots/10^6^ cells in SPF mice (n = 13), *p* = 0.037; rank-sum test). Notably, there was no difference in the MP4-specific IFN-γ response detected in the spleens of immunized conventionally housed (n = 4) and SPF (n = 9) mice (compare 547.5±346.4 to 442.6±364.4 spots per 10^6^ cells; *p* = 0.637; Student’s *t*-test).

**Figure 6:**
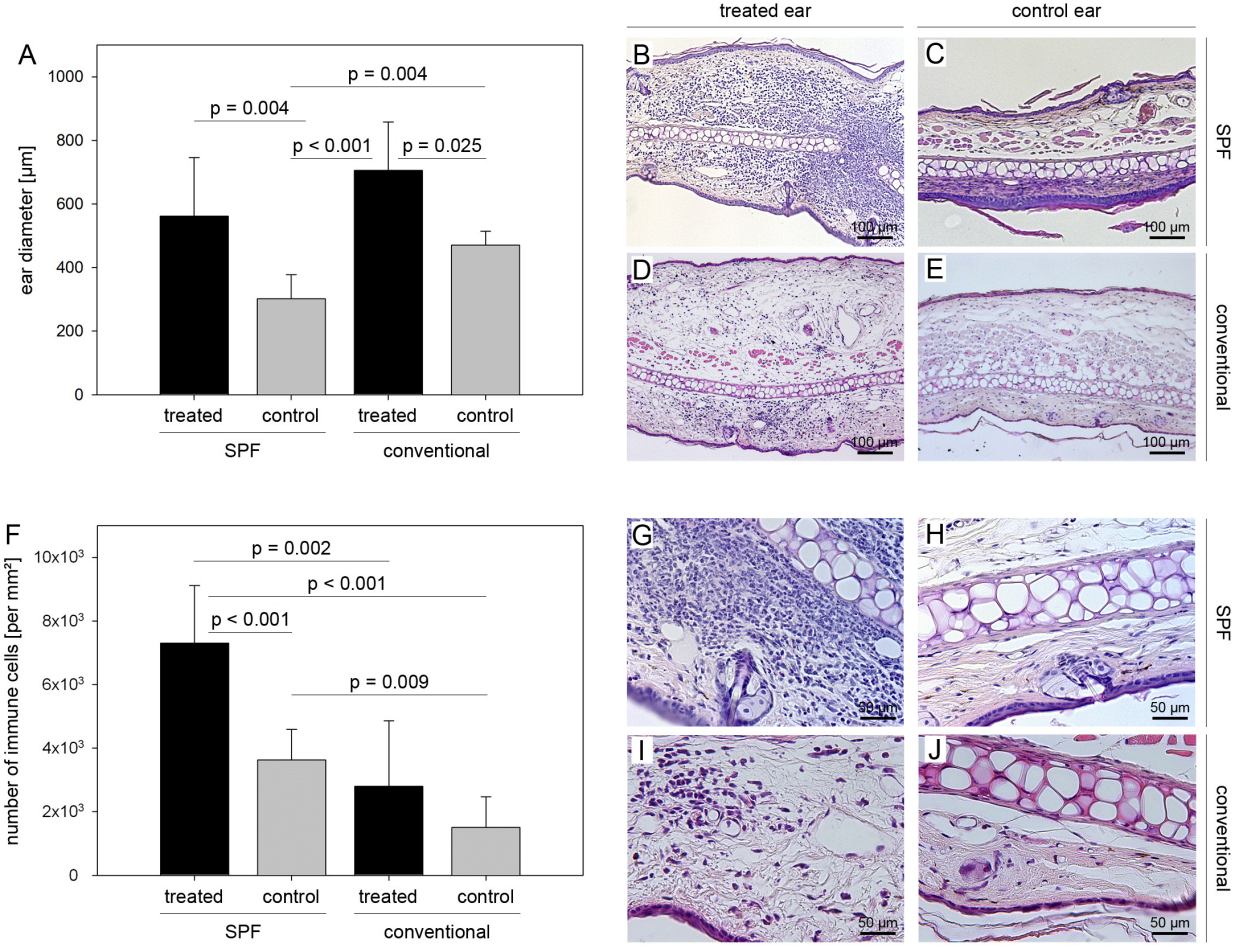
Attenuated DTH reaction in conventionally housed mice. Mice were housed either under conventional or SPF conditions and actively immunized with 200 µg MP4. On days 22–25 after immunization mice were challenged by intradermal ear injections with either 10 µg of MP4 or the irrelevant antigen OVA. After 24 h ear biopsies were taken and the extent of ear swelling (A–E) and cellular infiltration were determined (F–J) in each group. In the conventionally housed group n = 4 MP4- and n = 4 OVA-treated ears were analyzed. In the SPF group n = 5 MP4- and n = 6 OVA-treated ears were analyzed.

To further corroborate these results, we performed ear swelling assays that are typically used to assess DTH. Conventionally and SPF housed mice were immunized with MP4 and DTH assays were performed on days 22–25 after immunization as described in *Materials and Methods*. Results are shown in Figure 6. While the ear thickness was significantly increased in both conventionally and SPF housed mice that received MP4 compared to control injected mice (Figure 6A–E), only SPF mice showed a significant increase in the numbers of infiltrating cells/mm^2^ (Figure 6F–J). Numbers increased from 3626.77±967.94 in OVA- to 7301.07±1812.17 cells/mm^2^ in MP4-injected ears (*p*<0.001; Student’s *t*-test). In conventionally housed mice there was no significant difference between MP4- and OVA control injected ears (2801.58±2054.89 *versus* 1513.90±953.90 cells/mm^2^; *p* = 0.299; Student’s *t*-test).

## Discussion

The role of commensal microbiota in the etiology of MS is a recently emerging topic that is subject of debate. Using a spontaneous mouse model of MS it has been suggested that the commensal gut flora expands and activates CNS-reactive T cells, which subsequently recruit autoantibody producing B cells [2]. These data fit well into the concept that there are dietary factors involved in the development of MS and other autoimmune diseases. Among these factors are the vitamin D status, polyunsaturated fatty acids, milk proteins, gluten and probiotics [22]. In a recent publication it was reported that supplementation with probiotics improved disease activity and the inflammatory status in patients with rheumatoid arthritis (RA) [23] and the administration of a probiotic mixture exerted a therapeutic effect on EAE that was mediated by IL-10 [24]. Thus, microbiota cannot solely be regarded as a generalized disease causing agent. Rather a state of “dysbiosis” has been implied to have disadvantageous effects on autoimmune conditions such as inflammatory bowel disease (IBD), RA or diabetes mellitus type 1 (T1D), due to the initiation of an inflammatory cascade with subsequent local tissue disruption and clinical disease [25]–[27]. Along these lines, it has been demonstrated that the inflammasome pathway plays a key role in maintaining health. The knockout of inflammasome-related genes in mice resulted in the proliferation of *Prevotella* and TM7 phylotypes and the exacerbation of colitis [28].

In the study presented here we show that the development of EAE was diminished in mice that were kept under conventional housing conditions and carriers of *Staphylococcus spp.*, *Lactobacillus spp.*, *Streptococci, E. coli* and *Enterococcus spp.* in the gastrointestinal tract. Conventionally housed mice were characterized by an elevated immunoglobulin production in the Peyer’s patches. In addition, the size of the germinal centers within the Peyer’s patches in unimmunized control animals was comparable to the post-immunization status in the SPF mice. Immunization did not lead to a further increase in germinal center size in the conventionally housed mice. It is tempting to speculate that there is a relationship between the attenuation of disease development and an elevated baseline gut immune response in these mice. Decades ago it was shown that there was an inverse correlation between hay fever and the number of older siblings [29]. It then became widely acknowledged that certain infectious agents had a negative effect on the later development of allergies. Subsequently, a similar relationship was proposed for autoimmune diseases such as MS or T1D [30] and studies demonstrated a positive correlation between the sanitary conditions and the development of both T1D [31] and MS [32]. In addition, in a cohort of patients with MS that presented with parasitic infections a lower number of relapses and increased levels of IL-10 and TGF-β were observed [33]. In animal models, the development of T1D showed a direct association with the sanitary conditions of the animal facility in the sense that the infectious burden was inversely correlated with the disease incidence [30], [34]. Similarly, in EAE the application of mycobacteria was capable of preventing disease outbreak [35]. There are several mechanistic explanations how infections could protect against autoimmunity. One likely hypothesis deals with the concept of antigenic competition driving the affinity maturation of the secondary T cell response. It is widely acknowledged that immune responses, which are triggered by distinct antigens and occur simultaneously inhibit each other [36]–[38] and it was demonstrated that high-affinity T cells can out-compete T cells of lower affinity during a response to antigenic challenge *in vivo* [38]. This phenomenon was accompanied by the loss of antigen from the surface of antigen presenting cells, which was induced by high-affinity T cells and favored the activation of these cells. Further mechanisms involve the competition of leukocytes for cytokines and growth factors [39]. There are also other hypotheses that could be taken into account such as toll-like receptor (TLR) stimulation. In a model of spontaneous T1D, treatment with TLR agonists before the onset of disease completely abrogated disease progression [40]. However, the mechanisms of protective TLR stimulation are still not well defined [39].

Both cross-reactivity and antigenic competition between gut bacteria and CNS antigens may be important mechanisms involved in the development of EAE. It has recently been shown that antibiotic treatment that caused a reduction of commensal gut bacteria led to a reduction of disease activity in EAE. As a consequence, treatment of patients with MS and other autoimmune disorders was suggested as a reasonable option [41]. However, in follow-up studies, it was demonstrated that antigens from particular bacteria such as *B. fragilis* could also have a protective effect [42]. It was suggested that the presence or absence of polysaccharide A might be decisive for beneficial or harmful outcomes of EAE [42]. Also in MS not the mere presence of gut microbiota could be a key criterion for the development of or the protection against the disease, but a state of dysbiosis in favor of an autoimmune response directed against CNS antigens. It will be interesting to find out if future studies can achieve a subdivision of gut bacteria into pathogenic and protective strains to orchestrate treatment accordingly. The restoration of a balanced gut microflora might present a novel therapeutic strategy for MS and this concept is not only supported by the present study, but also underlines the possible influence of dietary factors. Concerning the results obtained in the animal model, future studies will have to address the question whether the involvement of gut bacteria is dependent on the genetic background of the mice, the antigen and the protocol used for EAE induction.

## Supporting Information

Checklist S1 **The ARRIVE Guidelines Checklist.**
(DOC)
